# The role of vitamin K in the prognosis of patients with hepatocellular carcinoma: a systematic review and meta-analysis

**DOI:** 10.3389/fonc.2026.1765445

**Published:** 2026-03-18

**Authors:** Lang Guo, Yaqiong Wang, Zhenkun Tan, Tingli Lu, Zha Peng, Hai Huang

**Affiliations:** 1 Wuming Hospital Affiliated to Guangxi Medical University, Nanning, Guangxi, China; 2 Graduate Institute, Guangxi Medical University, Nanning, Guangxi, China; 3 Dongguan Hospital of Guangzhou University of Traditional Chinese Medicine, Dongguan, Guangdong, China

**Keywords:** adjuvant therapy, hepatocellular carcinoma, meta-analysis, progression-free survival, transarterial chemoembolization (TACE), vitamin K

## Abstract

**Objective:**

This meta-analysis aimed to evaluate the influence of vitamin K (VK) as an adjunctive therapy on the prognosis of patients with hepatocellular carcinoma (HCC), with a specific focus on its clinical value in non-resected individuals.

**Methods:**

We systematically retrieved Chinese and English databases, including PubMed and Embase, to select randomized controlled trials (RCTs) and cohort studies comparing VK combined with standard therapy versus standard therapy alone. Methodological quality was evaluated via the Newcastle-Ottawa Scale for cohort studies and the Cochrane Risk of Bias tool (RoB-2) for RCTs. The primary endpoints were overall survival (OS) and progression-free survival (PFS). The secondary endpoint was recurrence risk. Pooled hazard ratios (HRs) and relative risks (RRs) were computed by fixed- or random-effects models. Subgroup analyses (surgery vs. transarterial chemoembolization [TACE]) and time stratification (12–48M) were conducted to explore heterogeneity sources.

**Results:**

Eleven studies involving 688 subjects were incorporated. The fixed-effects meta-analysis indicated that VK combined with standard therapy did not significantly improve OS (pooled HR = 0.77, 95% CI: 0.58–1.01). However, this combination prolonged PFS (pooled HR = 0.62, 95% CI: 0.47–0.82). Subgroup analysis based on PFS demonstrated more pronounced benefit in TACE-treated subjects (HR = 0.51, 95% CI: 0.34–0.77). Furthermore, VK combined with standard therapy reduced recurrence risk (pooled HR = 0.26, 95% CI: 0.15–0.46).

**Conclusion:**

Adjunctive VK improves PFS and reduces recurrence risk in HCC subjects, demonstrating particular benefit for those with unresectable tumors receiving TACE. No significant OS advantage was observed. Future investigations should optimize VK dosing and administration strategies and explore its potential synergy with immunotherapy.

**Systematic Review Registration:**

https://www.crd.york.ac.uk/PROSPERO/view/CRD420251106693, identifier CRD420251106693.

## 1 Introduction

Hepatocellular carcinoma (HCC) represents the sixth major cancer type and constitutes the fourth principal cause of cancer-related mortality (1). The prognosis for most HCC individuals remains poor, primarily due to late-stage diagnoses, which restrict the availability of curative treatments to a limited number of individuals. This scenario renders the management of HCC particularly challenging (2). Current therapeutic strategies for HCC require a comprehensive decision-making process based on staging, tumor burden, and hepatic function. These strategies include curative resection, ablation, transarterial chemoembolization (TACE), and systemic therapies (e.g., multi-targeted tyrosine kinase inhibitors and immune checkpoint inhibitors), as well as liver transplantation (3). Sorafenib was the only initial systemic treatment for HCC approved by the Food and Drug Administration for a decade. However, its efficacy has not been satisfactory (4). For individuals with unresectable or inoperable tumors, TACE is widely recognized as a first-line locoregional treatment strategy. Nonetheless, TACE’s effectiveness is significantly influenced by factors such as tumor biological behavior, hepatic reserve, and the frequency of treatment. This results in a common occurrence of recurrence and advancement (5). Notably, the TACE-induced ischemic and hypoxic microenvironment can trigger a ‘rebound’ of angiogenesis and invasion-related pathways. Such a rebound leads to suboptimal short-term outcomes or rapid progression in some patients. This underscores the urgent need for safe and accessible adjunctive or maintenance therapies to consolidate the benefits of TACE and delay disease progression (5).

Vitamin K (VK) serves as a cofactor for gamma-glutamyl carboxylase, essential for the activation of various VK-dependent proteins (6). Beyond its classical role in coagulation regulation, VK is also involved in multiple physiological processes, including bone metabolism, vascular homeostasis, and cellular stress responses (7). Its potential value in hepatology and oncology has garnered increasing attention. In the context of HCC, multiple biological connections exist between VK and tumors. On one hand, HCC individuals often exhibit abnormalities in coagulation factor synthesis and hepatic dysfunction. This suggests that VK supplementation may enhance the internal environment and provide ‘low-toxicity, foundational’ support for antitumor therapies (8). On the other hand, experimental and early clinical studies indicate that VK may inhibit tumor cell proliferation, induce apoptosis, and cause cell cycle arrest (9), as well as intervene in redox imbalance and associated signaling pathways (10). Additionally, VK has demonstrated some activity in reducing abnormal coagulation-related tumor markers (11). VK formulations, whether administered orally or via injection, are convenient, cost-effective, and exhibit good safety profiles. These advantages make them feasible and acceptable for long-term adjunctive or maintenance therapy in real-world populations.

In recent years, clinical evidence regarding “whether VK improves the prognosis of HCC patients” has gradually accumulated. This encompasses various scenarios such as postoperative adjuvant therapy, consolidation after ablation, combination with TACE, and integration with systemic treatment. However, previous studies have exhibited limitations in sample size, study design, staging of enrollment, types and doses of VK, combination regimens, and efficacy endpoints, leading to inconsistent conclusions. Among the unresectable HCC population in particular, evidence concerning “whether VK plus TACE can prolong progression-free survival (PFS) or overall survival (OS), reduce recurrence risk, or enhance objective response or disease control” remains fragmented. This evidence lacks systematic integration and quantitative assessment guided by clinical decision-making. This evidence gap directly impacts the mid-stage HCC population primarily treated with TACE, as well as inoperable patients seeking to delay progression with safe, long-term, affordable strategies.

As early as 2012 and 2013, systematic reviews and meta-analyses reported on the efficacy and safety of VK in adjuvant therapy following HCC resection (12, 13). The meta-analyses incorporated seven studies and suggested a potential delay in recurrence and an increase in disease-free survival rates. Nevertheless, these studies did not include new literature published in the past decade, particularly overlooking the non-surgical population. Thus, the present updated meta-analysis was conducted to comprehensively evaluate the efficacy and safety of VK in HCC individuals. Subgroup analyses were also implemented to explore VK’s clinical application value in ‘unresectable HCC patients’. The findings may provide more reliable evidence-based support for adjunctive therapy in this population.

## 2 Methods

Adhering to the Preferred Reporting Items for Systematic Reviews and Meta-analyses (PRISMA) guidelines (14), this review was prospectively registered in the International Prospective Register of Systematic Reviews platform (CRD420251106693) to ensure rigor and transparency.

### 2.1 Eligibility criteria

Studies that met the analytical criteria were defined using the Population, Intervention, Comparison, and Outcome (PICO) framework (15): Population (P): All individuals diagnosed with HCC per imaging or pathological standards recognized by guidelines, including those who underwent curative treatments (e.g., resection, ablation, or transplantation) and those with unresectable or inoperable tumors. Intervention (I): Any VK formulation alone or in conjunction with standard treatments (e.g., TACE, ablation, or systemic therapies), with no restrictions on administration route, dosage, or duration. Comparison (C): Standard treatment without VK supplementation or placebo, ensuring comparability of background treatment between the control and intervention groups, aside from VK. Outcome (O): The primary outcomes were OS and PFS. Safety outcomes included serious adverse events (SAEs). Hazard ratios (HR) and their 95% confidence intervals (95% CIs) were extracted for survival data, while binary outcomes could yield relative risks (risk ratio [RR]) or odds ratios (OR) along with their 95% CIs, or directly extract the number of events and total participants in each intervention group. Study design (S): Randomized controlled trials (RCTs) or prospective/retrospective cohort studies.

Exclusion criteria: (i) studies with duplicate reports; (ii) meta-analyses, reviews, or commentaries; (iii) letters or editorials; (iv) animal studies or mechanistic non-human research; (v) case reports; (vi) literature not in Chinese or English; (vii) conference abstracts with insufficient registration details or information.

### 2.2 Literature retrieval

Databases including the Cochrane Library, Embase, PubMed, Web of Science, China National Knowledge Infrastructure, Wanfang Data, VIP, and Chinese Biomedical Literature Database were retrieved up to July 16, 2025. The search strategy employed a combination of subject terms (e.g., MeSH, Emtree) and free-text terms, incorporating HCC-related terminology such as ‘Carcinoma, Hepatocellular’, ‘hepatocellular carcinoma’, ‘HCC’, ‘liver cancer’, ‘liver neoplasms’, and VK-related terms such as ‘Vitamin K’, ‘phylloquinone’, ‘menaquinone’, ‘menatetrenone’, etc. Boolean logic operators ‘OR’ and ‘AND’ were utilized to construct the search queries. Specifics are illustrated in **Supplementary Table 1**.

### 2.3 Literature screening

Literature screening was conducted by two independent investigators (Lang Guo and Yaqiong Wang). Initially, database search results were imported into EndNote for deduplication. Subsequently, titles and abstracts were subjected to a double-blind screening based on the predefined PICO criteria. Full texts of studies passing the initial screening were obtained. A thorough verification of the study population, intervention and control, outcomes, and study design was performed, with detailed documentation of exclusion reasons. For multiple reports from the same study cohort, the most comprehensive or longest-followed report was retained. If different reports provided complementary information, they were merged into a single study to avoid duplication. Discrepancies were resolved through discussion, and if necessary, adjudicated by a third investigator (Zhenkun Tan).

### 2.4 Information collection

Data were collected using a pre-structured Excel form. The extracted information comprised study characteristics (design, duration, sample size, and country of origin), patient demographics (sex, age, comorbidities, medication history, curative measures undertaken, and surgical status), recurrence status, and clinically relevant outcomes along with their effect sizes and 95% CIs (outcomes included PFS, OS, recurrence rates, etc.) (**Supplementary Table 2**). Information collection, tabulation, and verification were executed by two independent investigators (Lang Guo and Yaqiong Wang). Discrepancies were resolved through discussion, and if necessary, adjudicated by a third investigator (Zhenkun Tan).

For original studies directly reporting the results of Cox regression, we extracted HRs along with their corresponding CIs. For studies reporting relative risks of events between intervention groups at specific time points, RRs and their CIs were extracted. When only event rates at a given time point were reported, these were first converted into RRs with associated CIs before extraction. Owing to differences in statistical interpretation and calculation methods between HR and RR, separate meta-analyses were conducted for each metric.

### 2.5 Quality evaluation

The quality of studies was evaluated by two investigators (Lang Guo and Yaqiong Wang) via a standardized assessment tool. For RCTs, the Cochrane risk of bias assessment tool (RoB-2) was employed (16). The two reviewers independently assessed five domains: randomization process, deviations from intended interventions, missing outcome data, outcome measurement methods, and selection of reported results. The RoB for each domain was rated as ‘low risk’, ‘some concerns’, and ‘high risk’. If all domains were rated as ‘low risk’, the overall RoB was considered ‘low’. If some domains were rated as ‘some concerns’ without any ‘high risk’ domains, the overall risk was classified as ‘some concerns’. If any domain was rated as ‘high risk’, the overall risk was deemed ‘high’. For cohort studies, the Newcastle-Ottawa Scale was utilized. This instrument encompasses ‘sample selection’ (four stars), ‘comparability’ (two stars), and ‘outcome/exposure assessment’ (three stars) for rating. A rating of Good, Fair, and Poor corresponds to seven stars and above, four to six stars, and four stars or below, respectively. In cases of inconsistencies, a third investigator (Zhenkun Tan) facilitated a re-evaluation of the original texts and discussions to reach a consensus.

### 2.6 Data analysis

The primary outcome measures were OS, PFS, and recurrence rates. For studies that met inclusion criteria but did not directly report HRs, survival data were extracted from Kaplan-Meier survival curves using Engauge Digitizer to reconstruct HR estimates and their variances. Heterogeneity was examined via the *I²* statistic, defined as *I²* values exceeding 50%.

Stata 15.1 was applied for all statistical analyses. Inter-study heterogeneity for each outcome was quantified using I² (ranging from 0 to 100%). A random-effects model was employed for pooling when substantial heterogeneity was noted (I² > 50% and P < 0.05). Otherwise, a fixed-effects model was utilized. For outcomes with more than two studies, a leave-one-out sensitivity analysis was performed to test robustness. Subgroup analyses were conducted based on predefined treatment strategies (TACE group and surgical group) and time stratification by recurrence intervals (12M, 24M, 36M, 48M, and unspecified time points). Five studies were included in the time stratification analysis.

## 3 Results

### 3.1 Literature screening results

**Figure 1:**
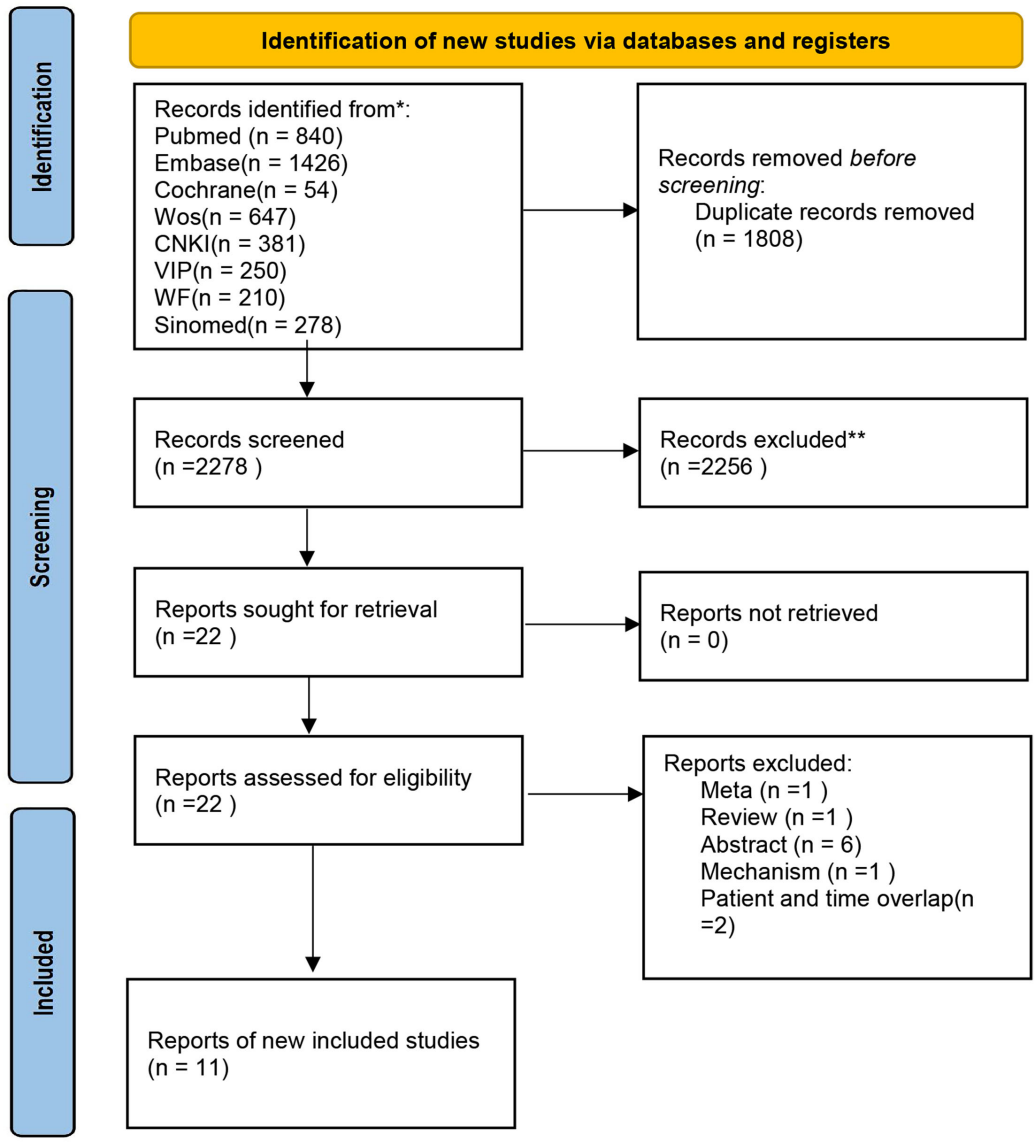
Prisma flowchart.

Database search yielded 4,086 records, which were initially imported into EndNote for deduplication. This procedure resulted in the removal of 1,808 duplicate entries. Then, an initial screening of titles and abstracts was conducted based on the predefined PICO criteria and exclusion principles. This screening led to the exclusion of 196 reviews or meta-analyses, 63 commentaries or letters, 177 animal or mechanistic studies, 180 case reports, 68 non-Chinese or non-English articles, and 18 registration protocols, as well as 325, 1,107, eight, and 115 studies failing to meet the population, intervention, outcome, and study design criteria, respectively. The remaining 22 studies were subjected to full-text evaluation. Eleven studies were excluded: one was a meta-analysis; one was a review; six were conference abstracts only; one was a mechanistic investigation; and two studies utilized overlapping cohorts from the same authors. Ultimately, 11 studies were incorporated for quantitative analysis (17–27), as detailed in **Figure 1**.

### 3.2 Basic information and quality evaluation

This systematic review included 11 studies published between 2006 and 2025 from three countries. Four of the studies were conducted in China, six in Japan, and one in India. The total sample size was 688 subjects, with individual study sample sizes ranging from 31 to 101 individuals. The patients’ ages varied from 25 to 89 years old. The majority were middle-aged or older individuals and male.

The intervention consisted of VK supplementation, which was primarily utilized as an adjunctive treatment alongside standard therapies such as TACE, sorafenib, usual treatment, or postoperative management. The intervention duration varied from short-term to long-term. The control group mainly received standard treatment alone (e.g., TACE alone, sorafenib alone, usual treatment, or without VK). The eligible studies consisted of nine RCTs and two cohort studies.

**Figure 2:**
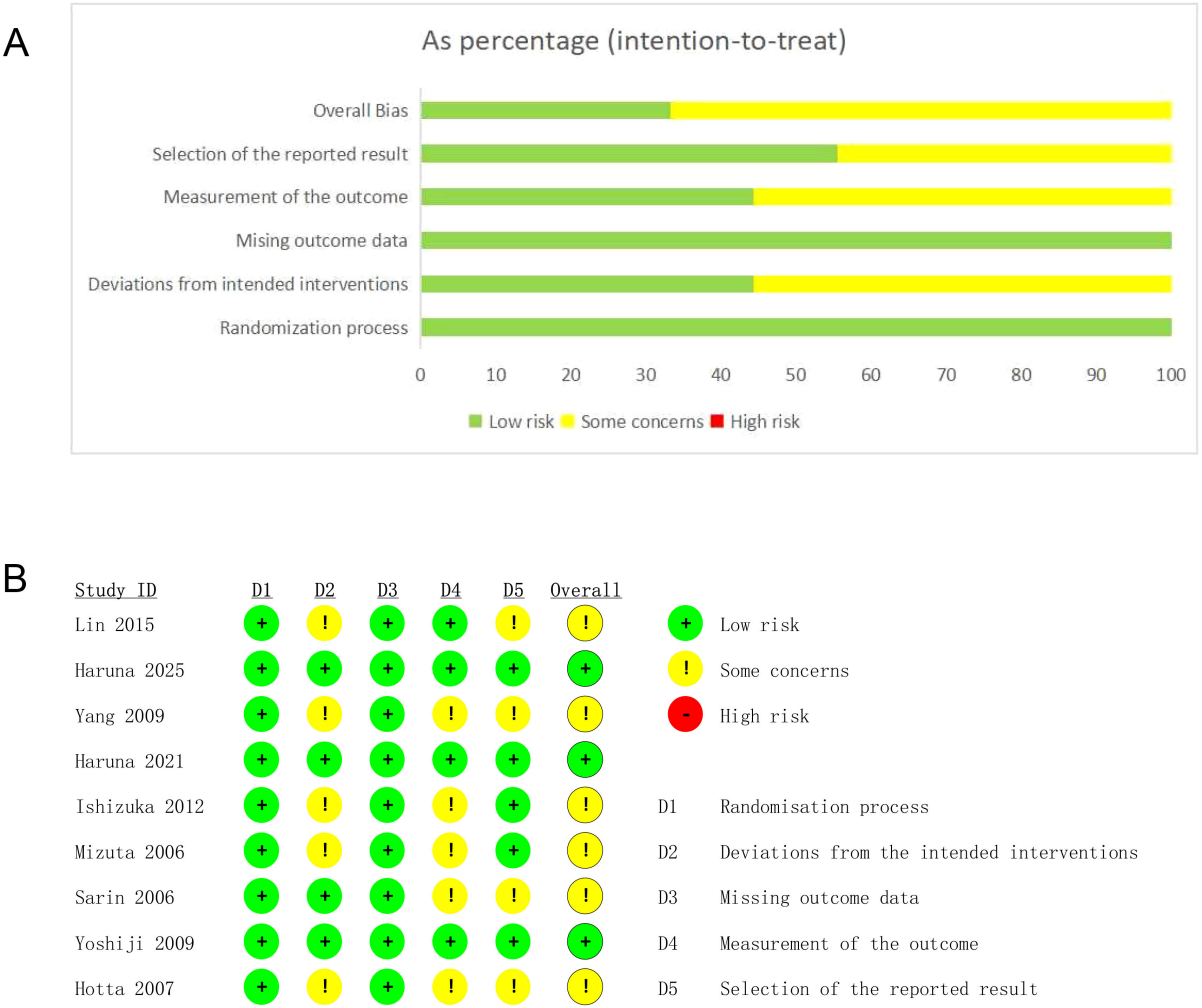
**(A)** ROB2 evaluation of RCTs-1; **(B)** ROB2 evaluation of RCTs-2.

All nine RCTs were evaluated as having no high risk. Three studies were rated as having a low risk, while the remaining six studies were classified as having some concerns. These concerns primarily involved deviations from intended interventions and outcome assessments. Specific differences were attributed to uncertainty or lack of blinding (**Figure 2**).

Two cohort studies were included, both of which were rated as having a low risk of bias and an overall quality score of seven (**Table 1**).

**Table 1:** NOS evaluation of cohort studies.

The surname of the first author	Year of publication	1) Representativeness of the exposed cohort	2) Selection of the non-exposed cohort	3) Ascertainment of exposure	4) Demonstration that outcome of interest was not present at start of study	5) Comparability of cohorts on the basis of the design or analysis (It can be 0 points, or 1 point, or 2 points)	6) Assessment of outcome	7) Was follow-up long enough for outcomes to occur	8) Adequacy of follow up of cohorts	Total	Grade
Hosho	2008	Y	Y	N	Y	NR	Y	Y	Y	7	good
Chen	2019	Y	Y	N	Y	NR	Y	Y	Y	7	good

### 3.3 Data analysis results

#### 3.3.1 OS

**Figure 3:**
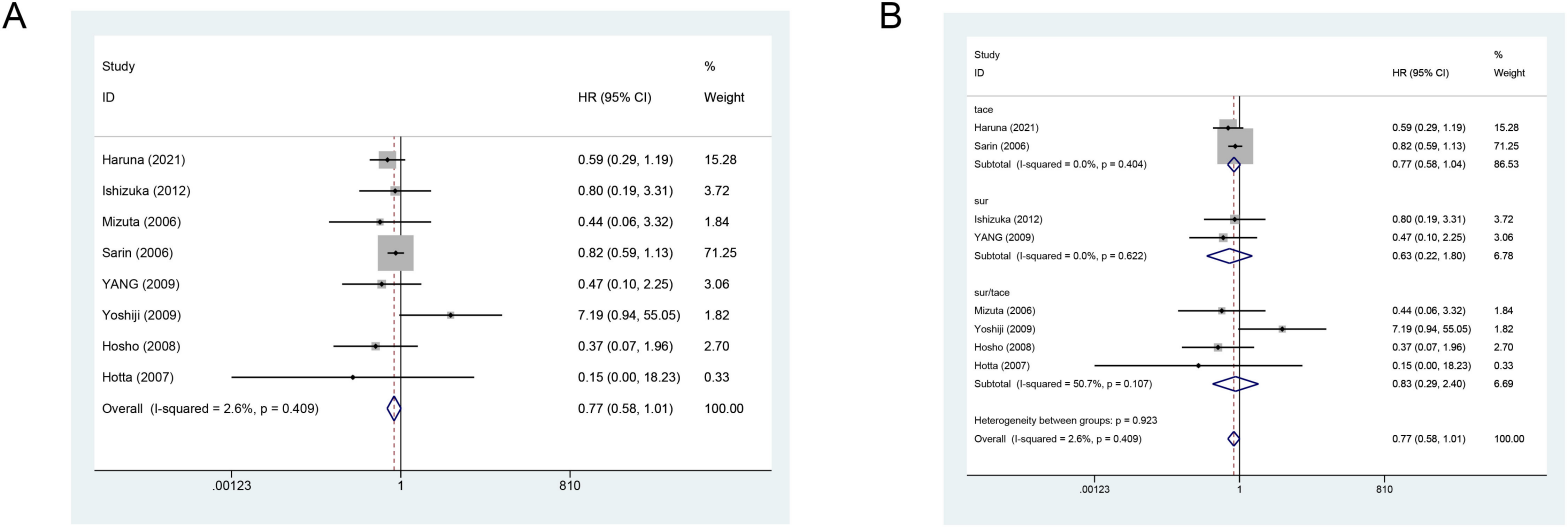
**(A)** Forest plot showing the effect of vitamin K combination therapy on overall survival (OS) in HCC patients. **(B)** Forest plot showing the effect of vitamin K combination therapy on OS in HCC patients receiving transarterial chemoembolization.

Eight studies reported the impact of VK on OS in HCC subjects. All original study results indicated no significant effect of VK on OS. The fixed-effects meta-analysis revealed that the concurrent use of VK with standard treatment did not improve OS (combined HR = 0.77, 95% CI: 0.58–1.01) (**Figure 3A**). The leave-one-out sensitivity analysis demonstrated the robustness of these results (**Supplementary Figure 1**).

Subgroup analysis (TACE group vs. surgical group) indicated that adding VK did not reduce the mortality risk in the surgical group (HR = 0.63, 95% CI: 0.22–1.80), nor did it in the TACE group (**Figure 3B**).

#### 3.3.2 PFS

**Figure 4:**
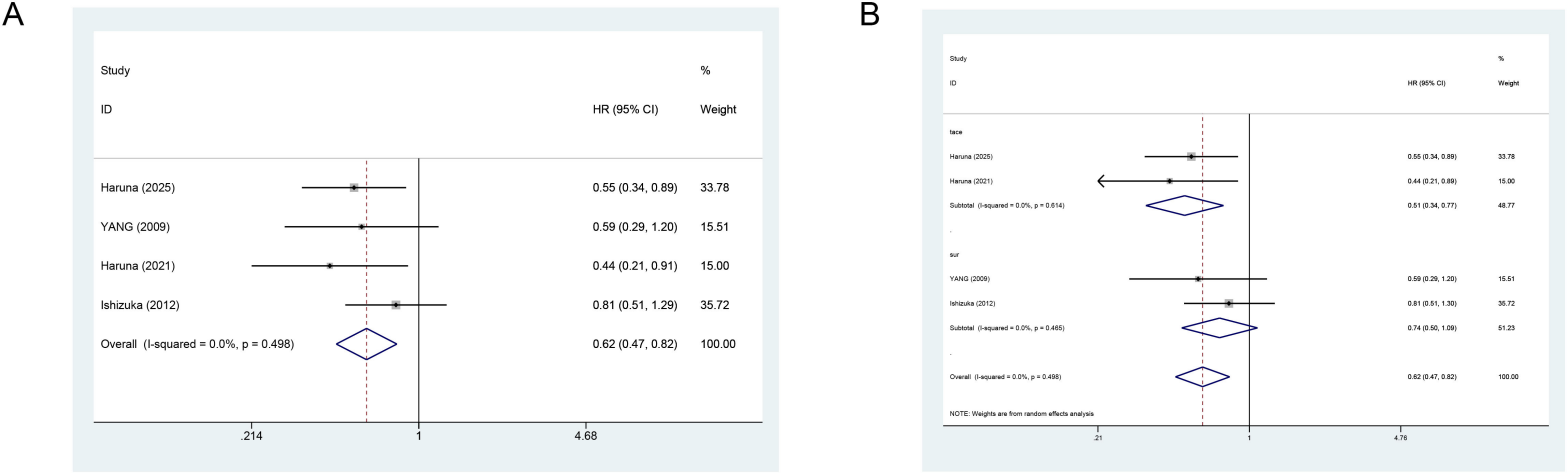
**(A)** Forest plot showing the effect of vitamin K combination therapy on PFS in HCC patients; **(B)** Forest plot showing the effect of vitamin K combination therapy on PFS in HCC patients receiving transarterial chemoembolization.

Four studies assessed the effect of VK on PFS (**Figure 4A**). All eligible original studies suggested that VK supplementation effectively reduced the recurrence risk in HCC individuals. The fixed-effects meta-analysis revealed that the VK combined with standard treatment improved PFS (combined HR = 0.62, 95% CI: 0.47–0.82). Sensitivity analysis using the leave-one-out method confirmed the robustness (**Supplementary Figure 2**).

Subgroup analysis showed that VK significantly reduced the risk of disease progression in TACE patients (HR = 0.51, 95% CI: 0.34–0.77). Nonetheless, the surgical group exhibited no reduction in such risk with the addition of VK (HR = 0.74, 95% CI: 0.50–1.09) (**Figure 4B**).

#### 3.3.3 Recurrence risk

**Figure 5:**
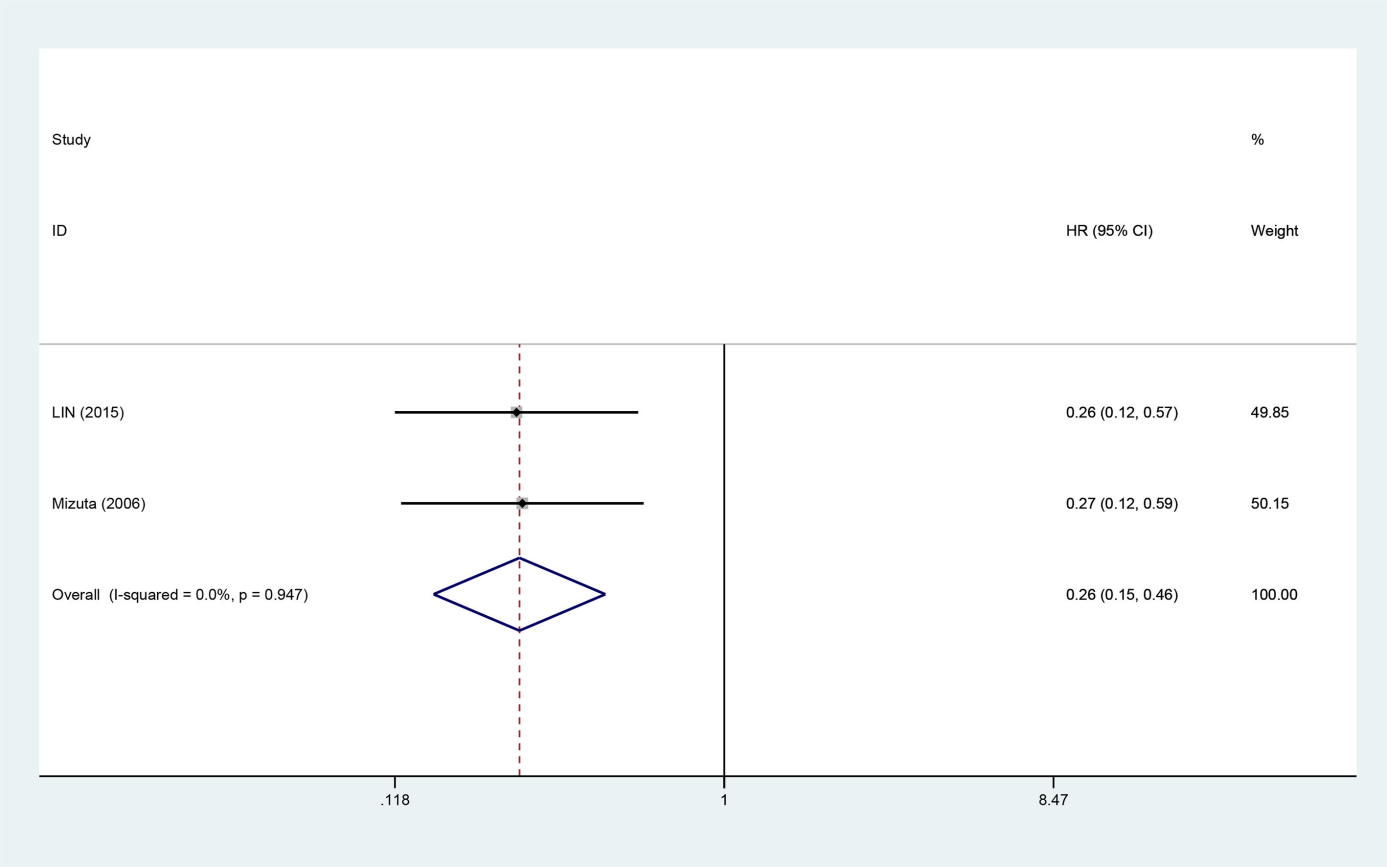
Forest plot showing the effect of vitamin K combination therapy on the recurrence risk in HCC patients.

Two studies evaluated the influence of VK on the recurrence risk (**Figure 5**). The fixed-effects meta-analysis indicated that the VK intervention group had significantly reduced recurrence risk relative to the control group (combined HR = 0.26, 95% CI: 0.15–0.46).

#### 3.3.4 Subgroup analysis for recurrence by follow-up duration

**Figure 6:**
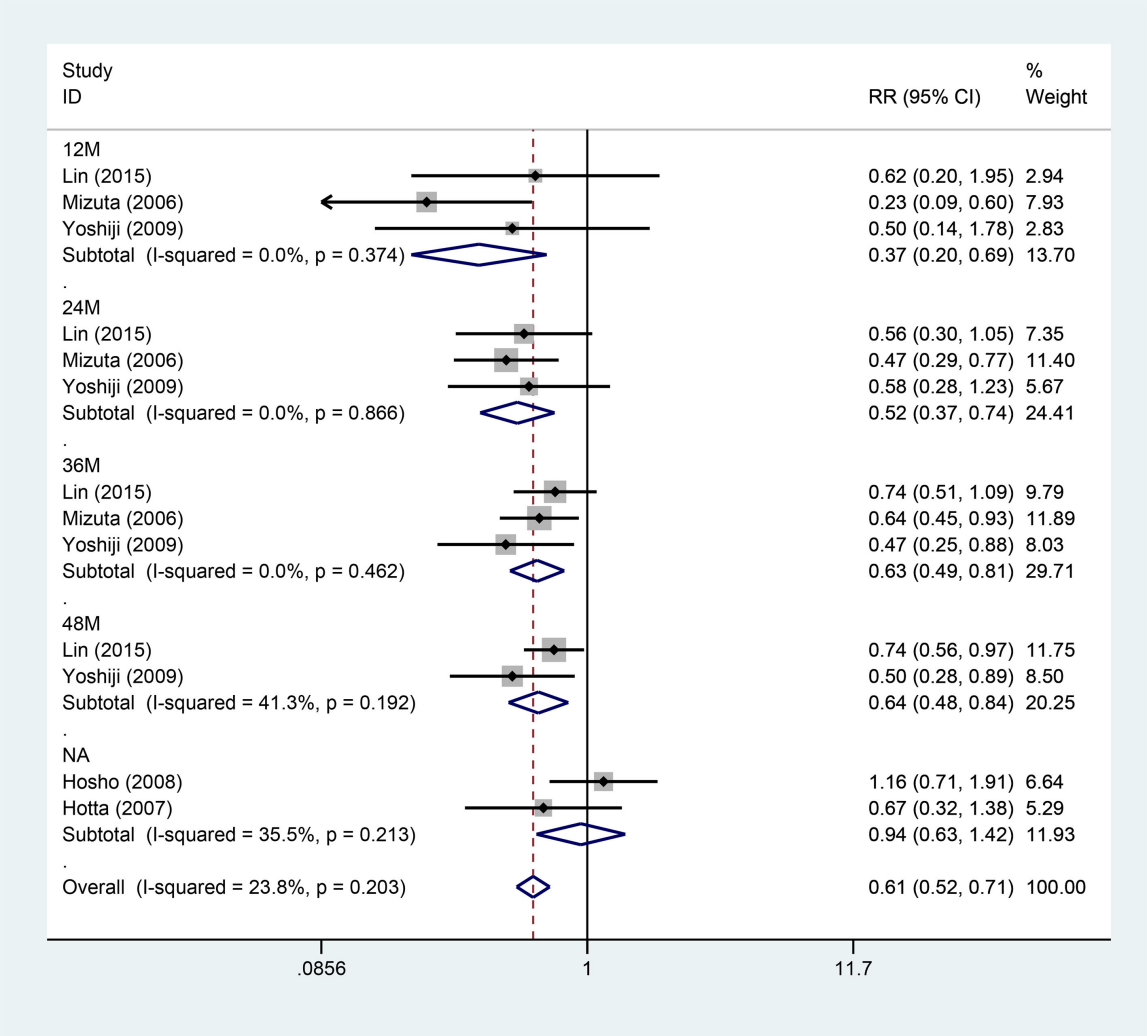
Forest plot of subgroup analysis by time for the effect of vitamin K combination therapy on the recurrence risk in HCC patients.

Five studies examined the impact of VK on the recurrence risk, with a stratified analysis based on follow-up duration (**Figure 6**). The five studies were divided into five subgroups: 12M, 24M, 36M, 48M, and unspecified time points according to the follow-up duration. Among these, three studies recorded follow-up up to 36M at the 12-month mark, while two studies followed up to 48M. Subgroup analysis indicated that within 12M of follow-up, VK notably reduced the recurrence risk, with three studies combined to show a pooled result of (RR [95% CI]: 0.37 [0.20-0.69]). At the 24-month follow-up, VK also reduced the recurrence risk, with three studies combined to yield a pooled estimate of (0.52 [0.37-0.74]). At the 36-month follow-up, VK still lowered the recurrence risk, with three studies combined to illustrate a pooled result of (0.63 [0.49-0.81]). At the 48-month follow-up, VK again reduced the recurrence risk, with two studies combined to produce a pooled estimate of (0.64 [0.48-0.84]). For the unspecified time point, the combined effect was not statistically significant (0.94 [0.63-1.42]).

The overall results of the subgroup analysis for recurrence by follow-up duration demonstrated that VK markedly reduced the recurrence risk in HCC subjects (combined RR = 0.61, 95% CI: 0.52–0.71, p < 0.001, I² = 23.8%, p = 0.203). The results at the 12, 24, 36, and 48-month time points all indicated significant benefits (RR < 1).

## 4 Discussion

The current meta-analysis reveals that the concomitant use of VK in HCC patients is related to improvements in PFS and recurrence risk, but not OS. For PFS, the TACE subgroup exhibited significantly reduced risk of disease progression with the addition of VK, whereas the surgical group failed to achieve a similar effect. Time-stratified subgroup analyses concerning recurrence indicated that the concurrent use of VK notably diminished the recurrence risk within a short follow-up duration (up to 48M). Despite the presence of some heterogeneity, robustness checks confirmed the stability of these findings.

The conclusions of our meta-analysis align with earlier meta-analyses (12) that reported a reduction in postoperative recurrence risk with VK. The current research updates existing studies and further investigates the influence of VK on the unresectable HCC population, employing time stratification to evaluate its effects on HCC recurrence. The findings suggest that the survival benefits of this therapy for patients undergoing curative surgery are limited. This is potentially linked to the biological characteristics of residual lesions following resection. The concurrent use of VK significantly reduces the recurrence risk in the short term. The combination strategy of VK with TACE demonstrates notable efficacy in unresectable HCC individuals, providing a new option for delaying disease progression in this population.

The VK combination strategy markedly enhances the PFS of HCC individuals while lowering recurrence risk. This effect may stem from VK2’s ability to inhibit IKK kinase activity, thereby preventing IκB phosphorylation and subsequently suppressing cyclin D1 expression (28). Additionally, VK2 inhibits PKC kinase activity and PKD1 activation. Such inhibition suppresses NF-κB activation (29), activates the protein kinase A/p53 pathway to inhibit the growth and invasion of HCC cells (30), and promotes p21 gene transcription to block the cell cycle while downregulating the expression of HCC-derived growth factor (31). Collectively, these pathways contribute to the suppression of tumor cell proliferation and the induction of cell cycle arrest.

Notably, patients with HCC commonly exhibit impaired liver function. Whether the supplementation of VK can indirectly enhance treatment tolerance through ameliorating hepatic function remains an important question to be investigated. Among the included studies, four reported the levels of ALT as mean ± standard deviation, two mentioned AST in baseline characteristics, and two provided data on total bilirubin. However, none reported the levels of albumin. This omission likely stems from the inherent characteristics of liver biochemical markers. Unlike primary endpoints such as PFS or OS, these markers are relatively indirect and susceptible to confounding factors. This susceptibility, combined with their inherent variability, may explain why many studies neglect longitudinal monitoring. Nevertheless, since ALT and AST are key biomarkers of hepatocellular injury, future clinical investigations should pay closer attention to them to better evaluate the potential therapeutic benefits of VK.

Subgroup analyses indicate that the combination strategy of VK with TACE was particularly effective, likely due to a synergistic effect between the two. The TACE-induced ischemic and hypoxic microenvironment upregulates pro-angiogenic factors (e.g., HIF-1α and VEGF), while VK2 selectively inhibits VEGFR2 phosphorylation (32), potentially blocking the common ‘angiogenesis rebound’ post-TACE and enhancing its anti-tumor efficacy. This observation is consistent with the significant improvements in PFS and reduced recurrence risk noted in TACE patients within the current meta-analysis. Conversely, the survival benefits of VK in subjects undergoing curative surgery appear limited. This is possibly because curative resection eliminates visible lesions, while residual micrometastatic foci may have lower sensitivity to VK or necessitate higher doses or prolonged interventions (33). Furthermore, the surgical procedure itself significantly reduces tumor burden. The present research did not demonstrate an improvement in OS attributable to VK. This may be explained by two factors. First, the included studies predominantly involved patients with intermediate to advanced HCC, in which the inherent aggressive nature and rapid progression of the tumors likely dominated survival outcomes. Second, many patients presented with coexisting cirrhosis or active hepatitis. Liver failure or complications from portal hypertension (e.g., variceal hemorrhage) may have been more significant causes of mortality than tumor progression (34). These competing risks may have diminished the direct impact of VK intervention on OS.

Biological features of micrometastases of HCC—such as dormancy and inadequate vascularization—may also underlie the limited efficacy observed with VK therapy (35). Conceivably, high-dose or prolonged administration of VK might effectively counteract residual postoperative micrometastases. This hypothesis should be validated in future investigations.

This research indicates that VK can significantly improve PFS and reduce short-term recurrence risk in patients with intermediate-stage or advanced unresectable HCC undergoing TACE. The underlying mechanism may involve suppression of post-TACE angiogenic rebound and regulation of the tumor cell cycle (32). Nonetheless, VK supplementation failed to enhance OS and provided limited survival benefit for patients receiving curative resection. These findings suggest that the therapeutic efficacy of VK is potentially influenced by treatment modality (curative surgery or TACE) and tumor biological behavior.

The potential value of VK in the comprehensive treatment of unresectable HCC, particularly as a synergistic and maintenance therapy in conjunction with TACE, warrants clinical attention. Our analysis suggests that VK formulations could be an effective adjunctive strategy for TACE treatment. Combining VK with conventional HCC therapies has advantages, such as being more cost-effective than many targeted or immunotherapeutic agents. This alleviates the economic burden on patients and healthcare systems. Furthermore, existing clinical data support its favorable safety profile, demonstrating good tolerability with long-term oral administration and a very low incidence of SAEs. Patients primarily experience mild and manageable side effects (19). The combination of VK’s high safety profile and low cost makes it a suitable option for patients requiring long-term maintenance therapy following TACE. VK has the potential to enhance patient survival outcomes, optimize treatment adherence, and improve overall quality of life.

The present meta-analysis indicates that a combined VK strategy significantly improved the prognosis of HCC individuals not receiving curative resection, particularly those undergoing TACE. This provides a basis for prognostic assessment with VK in this population. Prespecified subgroup analysis, stratified by treatment (surgery vs. TACE), further confirmed the marked efficacy of the VK-TACE combination in patients with unresectable HCC. These findings offer clinicians additional options for delaying disease progression, especially in the management of unresectable HCC.

Nonetheless, certain included RCTs may exhibit deficiencies and shortcomings in adherence to intended interventions and outcome measurements, resulting in an RoB in the evaluation outcomes. Additionally, there are regional and etiological biases since the majority of the included studies originated from Asia and focused on HBV-related HCC. These limitations constrain the generalizability of our conclusions, particularly regarding applicability to Western populations. Although follow-up durations were adequate in many studies, the eligible trials had relatively small sample sizes. This hinders a thorough evaluation for long-term risks and benefits of VK.

Moreover, the limited number of studies within the surgical subgroup may have resulted in insufficient statistical power. This potentially masks genuine therapeutic effects.

Regarding dosing protocols, varying doses of VK or treatment durations might contribute to inconsistent clinical outcomes. Future research should address the necessity and consistency of optimal regimens.

Emerging avenues warrant exploration in forthcoming studies. For instance, NAT10 stabilizes SMAD3 mRNA via the modification of ac4C, thereby driving the progression of HCC and activating the TGF-β signaling pathway. Targeting NAT10 for treating HCC represents another plausible mechanism influencing outcomes (36).

## 5 Conclusion

Our meta-analysis demonstrates that the concomitant use of VK as an adjunctive therapy in treating HCC is linked to improvements in PFS and recurrence risk, but not OS. For postoperative patients, adding VK does not markedly reduce the risk of disease progression. Future research should focus on optimizing VK dosing, exploring combination strategies with novel therapies (e.g., immunotherapy), and developing biomarker-based precision treatment models to potentially yield greater survival benefits for HCC patients.

## Data Availability

The original contributions presented in the study are included in the article/**Supplementary Material**. Further inquiries can be directed to the corresponding authors.
