# Supplementary material for: The role of vitamin K in the prognosis of patients with hepatocellular carcinoma: a systematic review and meta-analysis
Source: Front Oncol. 2026 Mar 18;16:1765445. doi: 10.3389/fonc.2026.1765445 (PMC13038519; doi:10.3389/fonc.2026.1765445)
Supplement: Supplementary file 1 [file DataSheet1.docx]

**Supplementary materials**

**
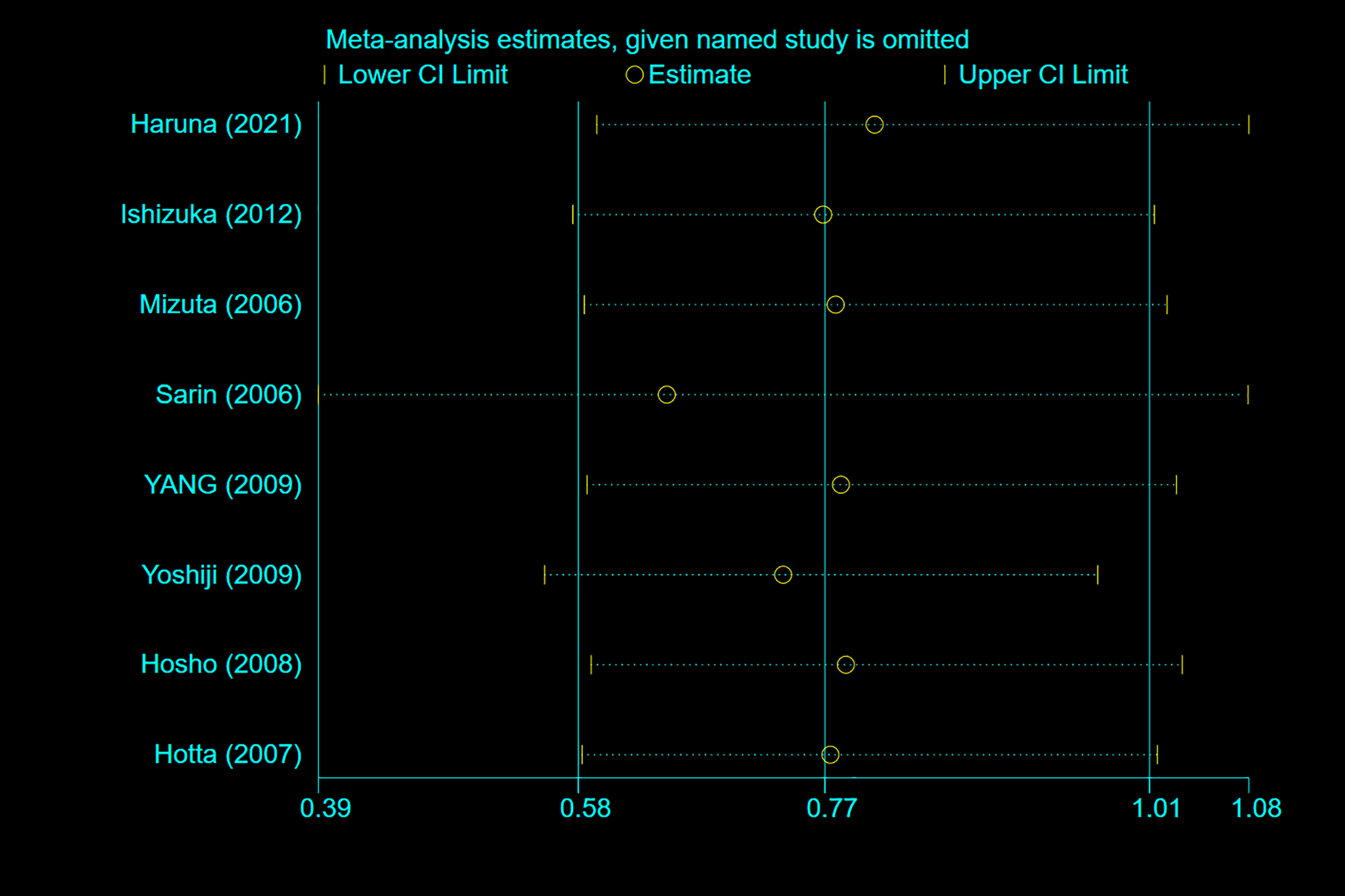
**

**Supplementary Fig. 1:** Sensitivity analysis of the effect of vitamin K combination therapy on overall survival (OS) in HCC patients.

**
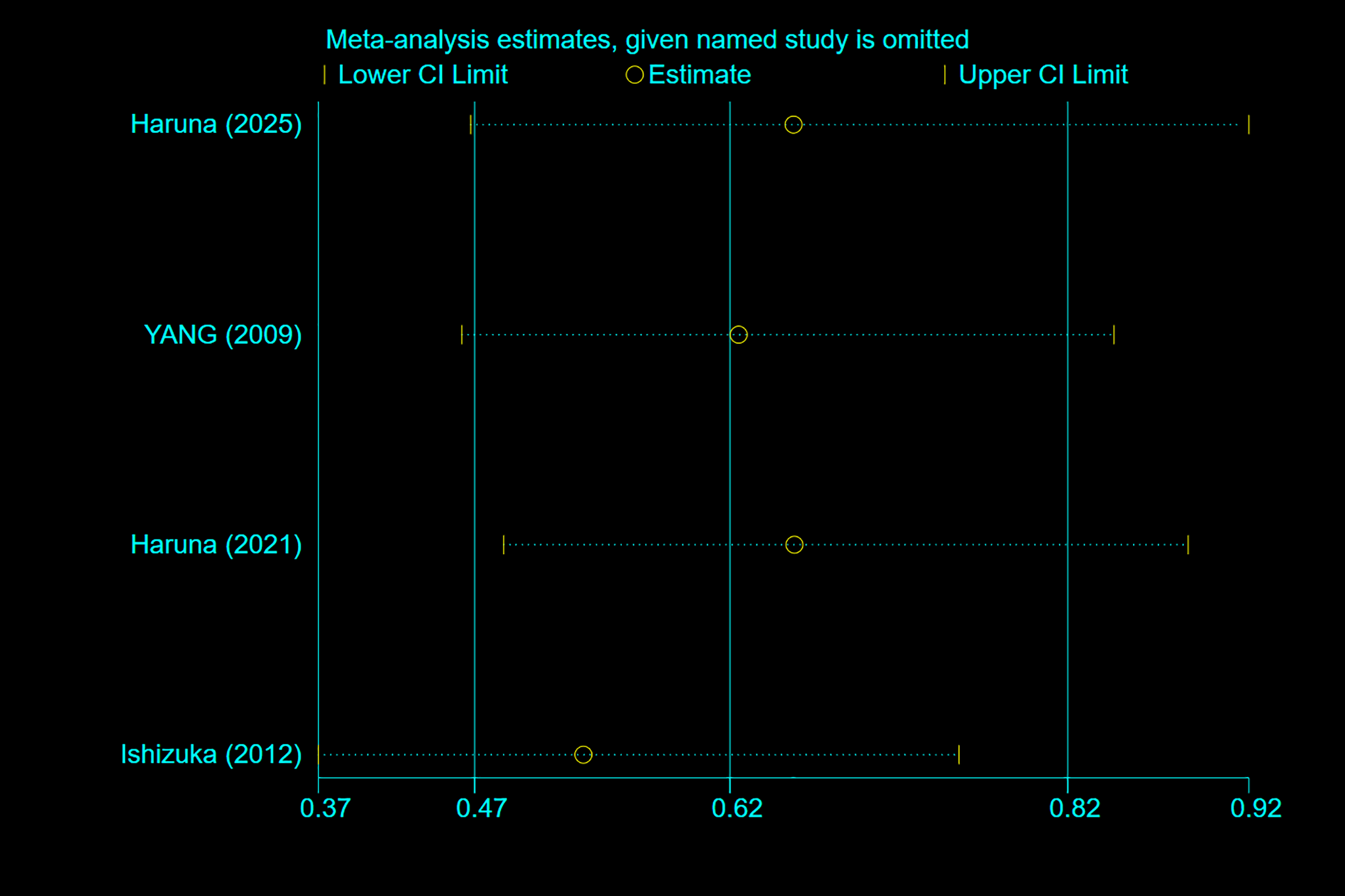
**

**Supplementary Fig. 2:** Sensitivity analysis of the effect of vitamin K combination therapy on PFS in HCC patients.

**Supplementary Table 1:** Search Results

| Pubmed | | |
| --- | --- | --- |
| # | Query | Results |
| 1 | Carcinoma, Hepatocellular | 115,519 |
| 2 | primary liver carcinoma" OR "malignant hepatoma" OR "Liver Cell Carcinomas" OR "Liver Cell Carcinoma" OR "liver carcinoma rupture" OR "liver carcinoma" OR "hepatomatous" OR "hepatomata" OR "Hepatomas" OR "hepatoma" OR "hepatocytic carcinoma" OR "hepatocyte carcinoma" OR "hepatocellular carcinomata" OR "Hepatocellular Carcinomas" OR "hepatocellular carcinoma" OR "hepatocarcinoma" OR "hepato cellular carcinoma" OR "hepato carcinoma" OR "hepatic cell carcinoma" OR "hepatic carcinoma" OR "carcinoma of the liver" OR "carcinoma in the liver" OR "Adult Liver Cancers" OR "Adult Liver Cancer | 171,817 |
| 3 | Vitamin K | 19,821 |
| 4 | vitamin K group" OR "vitamin K" OR "phytomenadione" OR "menaquinone" OR "menadione | 28,249 |
| 5 | ****(#1 OR #2) AND (#3 OR #4)**** | 840 |

| Embase | | |
| --- | --- | --- |
| # | Query | Results |
| 1 | ‘liver cell carcinoma’/exp | 242581 |
| 2 | (‘primary liver carcinoma' OR 'malignant hepatoma' OR 'Liver Cell Carcinomas' OR 'Liver Cell Carcinoma' OR 'liver carcinoma rupture' OR 'liver carcinoma' OR 'hepatomatous' OR 'hepatomata' OR 'Hepatomas' OR 'hepatoma' OR 'hepatocytic carcinoma' OR 'hepatocyte carcinoma' OR 'hepatocellular carcinomata' OR 'Hepatocellular Carcinomas' OR 'hepatocellular carcinoma' OR 'hepatocarcinoma' OR 'hepato cellular carcinoma' OR 'hepato carcinoma' OR 'hepatic cell carcinoma' OR 'hepatic carcinoma' OR 'carcinoma of the liver' OR 'carcinoma in the liver' OR 'Adult Liver Cancers' OR 'Adult Liver Cancer’):ti,ab,kw | 241402 |
| 3 | ‘vitamin K group’/exp | 38097 |
| 4 | (‘vitamin K group' OR 'vitamin K' OR 'phytomenadione' OR 'menaquinone' OR 'menadione’):ti,ab,kw | 40701 |
| 5 | ****(#1 OR #2) AND (#3 OR #4)**** | 1426 |

| Cochrane Library | | |
| --- | --- | --- |
| # | Query | Results |
| 1 | MeSH descriptor: [Carcinoma, Hepatocellular] explode all trees | 2820 |
| 2 | (‘primary liver carcinoma' OR 'malignant hepatoma' OR 'Liver Cell Carcinomas' OR 'Liver Cell Carcinoma' OR 'liver carcinoma rupture' OR 'liver carcinoma' OR 'hepatomatous' OR 'hepatomata' OR 'Hepatomas' OR 'hepatoma' OR 'hepatocytic carcinoma' OR 'hepatocyte carcinoma' OR 'hepatocellular carcinomata' OR 'Hepatocellular Carcinomas' OR 'hepatocellular carcinoma' OR 'hepatocarcinoma' OR 'hepato cellular carcinoma' OR 'hepato carcinoma' OR 'hepatic cell carcinoma' OR 'hepatic carcinoma' OR 'carcinoma of the liver' OR 'carcinoma in the liver' OR 'Adult Liver Cancers' OR 'Adult Liver Cancer’):ti,ab,kw | 14042 |
| 3 | MeSH descriptor: [Vitamin K] explode all trees | 873 |
| 4 | (‘vitamin K group' OR 'vitamin K' OR 'phytomenadione' OR 'menaquinone' OR 'menadione’):ti,ab,kw | 3186 |
| 5 | (#1 OR #2) AND (#3 OR #4) | 54 |

| Web of Science | |
| --- | --- |
| Query | Results |
| “primary liver carcinoma" OR "malignant hepatoma" OR "Liver Cell Carcinomas" OR "Liver Cell Carcinoma" OR "liver carcinoma rupture" OR "liver carcinoma" OR "hepatomatous" OR "hepatomata" OR "Hepatomas" OR "hepatoma" OR "hepatocytic carcinoma" OR "hepatocyte carcinoma" OR "hepatocellular carcinomata" OR "Hepatocellular Carcinomas" OR "hepatocellular carcinoma" OR "hepatocarcinoma" OR "hepato cellular carcinoma" OR "hepato carcinoma" OR "hepatic cell carcinoma" OR "hepatic carcinoma" OR "carcinoma of the liver" OR "carcinoma in the liver" OR "Adult Liver Cancers" OR "Adult Liver Cancer” | 182753 |
| "vitamin K group" OR "vitamin K" OR "phytomenadione" OR "menaquinone" OR "menadione" | 22815 |
| #1 AND #2 | 647 |

| CNKI | |
| --- | --- |
| Query | Results |
| （篇关摘：肝癌 + 肝恶性肿瘤 + 肝腺癌 + 肝肿瘤 + 肝细胞癌(精确)）AND（篇关摘："维生素K" + "vitamin k"(精确)） | 381 |

| VIP | |
| --- | --- |
| Query | Results |
| (((((题名或关键词=肝癌 OR 题名或关键词=肝恶性肿瘤) OR 题名或关键词=肝腺癌) OR 题名或关键词=肝肿瘤) OR 题名或关键词=肝细胞癌) AND (摘要="维生素K" OR 摘要="vitamin k")) | 250 |

| WF | |
| --- | --- |
| Query | Results |
| 题名或关键词:(肝癌 OR 肝恶性肿瘤 OR 肝腺癌 OR 肝肿瘤 OR 肝细胞癌) and 题名或关键词:("维生素K" OR "vitamin k") | 210 |

| SinoMed | |
| --- | --- |
| Query | Results |
| ( "肝癌"[常用字段:智能] OR "肝恶性肿瘤"[常用字段:智能] OR "肝腺癌"[常用字段:智能] OR "肝肿瘤"[常用字段:智能] OR "肝细胞癌"[常用字段:智能]) AND( ""维生素K""[常用字段:智能] OR ""vitamin k""[常用字段:智能]) | 278 |

**Supplementary Table 2:** Basic Information of the included studies

| General Information | | | Demographic information of population | | | | Intervention | | | | | Control | | | |  |  |
| --- | --- | --- | --- | --- | --- | --- | --- | --- | --- | --- | --- | --- | --- | --- | --- | --- | --- |
| First Author | Publication Year | Country | Sample Size | Age | Gender(M/F) | Description of disease severity | Name | Dose | Routine | Frequency | Duration | Name | Dose | Routine | Duration | outcome | study design |
| Chen | 2019 | CHINA | 31 | 48.0±10.5 49.6±10.3 54.3±8.7 | 23/8 | Patients with advanced HCC who visited the hospital between March 2016 and March 2017, met the diagnostic criteria for HCC according to the "Guidelines for the Diagnosis and Treatment of Primary Liver Cancer in China," and had lost the opportunity for surgery, were selected. | TACE+VK1 | 40 mg once | oral | once/d | 4 w after TACE | TACE group, VitK1 group | NA | oral | NA | Changes in serum DCP levels, tumor response rate (4 weeks after treatment) | Cohort study |
| Lin | 2015 | CHINA | 54 | 25~72 | （43/11） | Inclusion criteria: Age 20 and above, performance status according to the Eastern Cooperative Oncology Group (ECCG) score (0-2); compensated liver function (albumin ≥28 g/L; total bilirubin <2.0 μmol/L; prothrombin activity ≥40%). Exclusion criteria: Previous systemic or hepatic artery chemotherapy; extrahepatic metastasis; virological response to interferon therapy within 2 years; uncontrolled hepatic encephalopathy; refractory ascites or pleural effusion; post-gastrectomy, extensive gastrointestinal tract resection, or cholecystectomy; multiple co-existing conditions (severe cardiovascular, hematologic, or renal diseases); history of cancer other than liver cancer within 5 years; treatment with warfarin and vitamin K within 6 months; pregnant women, women about to give birth, or breastfeeding women. | In addition to conventional treatment, take vitamin K2 orally | 45 mg | oral | daily | 4Y | standard therapy | NA | NA | 4Y | Cumulative recurrence rate, cumulative survival rate | RCT |
| Haruna | 2025 | JAPAN | 101 | 53~89 | (71/30) | ≥ 18 years of age, ≤89 years of age, a life expectancy of ˃12 weeks, maximum tumor diameter of ≤10 cm, ≤2 prior TACE sessions, Eastern Cooperative Oncology Group performance status of 0 or 1, and Child-Pugh score of A to B7. | TACE+VK | 45 mg | oral | daily | NA | tace | NA | NA | NA | Objective response rate (ORR) (including complete response (CR), partial response (PR), stable disease (SD), and no progression (PD)?) PFS | RCT |
| Yang | 2009 | CHINA | 50 | 35~64 | （42/8） | All patients with primary hepatocellular carcinoma were diagnosed by pathological examination. All patients also had chronic hepatitis B, but no HCV infection was observed. No tumor recurrence was observed within one month of radical tumor resection. | Oral vitamin K2 | 45 mg | oral | daily | 36M | No use of vitamin K | NA | NA | NA | Cumulative survival rate, tumor-free survival rate | RCT |
| Haruna | 2021 | JAPAN | 44 | 72.±8.3 71.9 ± 8.9 | （35/9） | Patients with advanced HCC who had not received previous systemic therapy and had unresectable tumors with macro- scopic vascular invasion, extrahepatic spread, or transarterial chemoembolization failure were eligible. | Sorafenib +vitamin K | 15mg | oral | three times a day | NA | Sorafenib alone | 400mg | NA | NA | ORR (complete response (CR) or partial response (PR) lasting at least four weeks), disease control rate (maintaining CR, PR or stable disease (SD) for at least four weeks), overall survival (OS) and safety | RCT |
| Ishizuka | 2012 | JAPAN | 101 | 68.4±9 65.8±8.2 | （78/23） | Patients who had undergone curative hepatectomy for the first time under a preoperative diagnosis of HCC, and whose surgical specimens had been histologically confirmed to be HCC, were recruited for this study. | MNT | 45mg | oral | daily | 17~68M | non-MNT | NA | NA | 17~68M | Cumulative disease-free survival rate,OS | RCT |
| Mizuta | 2006 | JAPAN | 61 | 63.3 ⫾ 7.5(48-75);64.5⫾ 6.7(45-74) | (41/20) | Patients were diagnosed as free of HCC after surgical resection or percutaneous local ablation | Menatetrenone | 45mg | oral | daily | NA | Untreated group | NA | NA | NA | Cumulative recurrence rate, OS | RCT |
| Sarin | 2006 | INDIA | 42 | 27–72 | (33/9) | (i) HCC diagnosed as stage C (advanced) according to BCLC staging system; (ii) unresectable cancer, as assessed carefully by individ- ual experts; (iii) no recent (within the past 6 months) active treat-ment, including surgery, radiotherapy, chemotherapy, transarterial embolization, percutaneous ethanol injection or other regional treatment;  and (iv) invasive pattern as evidenced by vascular inva- sion or extracellular spread diagnosed by computed tomography (CT) or magnetic resonance imaging (MRI). | Vitamin K | high dose vitamin K3 infusion; and (ii) placebo. The vitamin K3 was administered by i.v. infusion of 50 mg/day with daily increase of dose by 50 mg for 6 days, followed by 20 mg i.m. | oral | twice a day | 2 weeks | Placebo | (i) high dose vitamin K3 infusion; and (ii) placebo. The vitamin K3 was administered by i.v. infusion of 50 mg/day with daily increase of dose by 50 mg for 6 days, followed by 20 mg i.m. twice a day for 2 weeks. | Intravenous injection | NA | Objective response rate (ORR) (including complete response (CR), partial response (PR), and objective response) (OS) | RCT |
| Yoshiji | 2009 | JAPAN | 87 | 51.1~71 | 52/32 | HCC patienst | TACE+VK | 45mg vk+4mg acei | oral | daily | 48mouths | NA | NA | NA | NA | Cumulative recurrence rate (no OS?) | RCT |
| Hosho | 2008 | Japan | 72 | 60-78 | 38/34 | HCC patienst | Vitamin K | 45 mg | oral | daily | NA | Non-treated control group | NA | NA | NA | Recurrence rate | Cohort study |
| Hotta | 2007 | JAPAN | 45 | 52~84 | （33/12） | HCC patients who have been cured | Vitamin K | 45 mg | oral | daily | NA | other type of drugs to treat osteoporosis | NA | NA | NA | Cumulative survival rate and recurrence rate | RCT |
